# Book Reviews

**Published:** 1817-08

**Authors:** 


### CRITICAL ANALYSIS
### *OF RECENT PUBLICATIONS,*
IN THE
### DIFFERENT BRANCHES OF PHYSIC, SURGERY, AND MEDICAL PHILOSOPHY.

---

*Transactions of the Medical Society of London.* Vol. I. Part II. 1817.

Art. I.—*On Epilepsy;* by JOSEPH ADAMS, M.D. &c. President of the Society.

THIS is the first paper, systematically written, that has been offered to our notice on this formidable disease. Our last Collectanea, therefore, consisted of a long extract, by which our readers will comprehend the general intent of the author. The remainder of the paper contains a minute description of the acute epilepsy. The treatment (bleeding and other evacuations before the expected period of a return of paroxysms) exactly accords with his opinion of the disease. Several cases are detailed; and, in all which have appeared under the form of acute epilepsy, the mode of treatment has been completely successful.

A few remarks follow on the chronic epilepsy, in which, with much candour, the author admits he has been as completely foiled, and seems sceptical in his opinion of the various remedies recommended by others. For the diagnostic distinction between the acute and chronic epilepsy, we must refer to the paper.

Art. II.—*Observations on the Treatment of Croup;* by HENRY BLEGBOROUGH, Esq. Surgeon.

The object of this practical and truly valuable paper is to shew, that, whatever may have been the success of other practitioners, the author has been disappointed in every attempt at arresting the progress of the disease, excepting by the assistance of mercury in large doses. Mr. Blegborough's meaning will be best expressed by the two following cases.

"Case V.—On the 13th of October, 1807, I was called to see —— Cave, a girl aged two years: she had the usual symptoms of catarrh, which continued without much variation till the morning of the 22d, when the cough, difficulty of breathing, and fever, were somewhat increased; the child had been frequently purged, and had taken lact. amygd. with nitre. On the 23d, in the morning, I was sent for early: the child had passed a very restless night, had much much fever, hoarse and difficult breathing, and ringing cough; in fact, all the symptoms of croup. Dissatisfied with the remedies which I had before employed, I wished, though with the same general intention of diminishing the force of the circulation in the first instance, and subsequently of effecting the removal of the collected mucus, if, in despite of every thing, as before, it should be formed, to employ a different agency; and, having seen the good effect of nauseating doses of antim. tartarizat. in keeping down the circulation, and in taming the violence of athletic men under the influence of maniacal attacks, I determined to rest my first intention upon this remedy, and to employ full vomiting in the after stage, if my patient should arrive at it. Accordingly, a solution of this medicine, in the proportion of gr. iij. to an ℥ of water, was given, to the effect of keeping up a continued and very distressing degree of nausea, from the morning of the 23d till about two o'clock P. M. of the 24th, during which time the circulation was very languid: there was, of course, no disposition to take sustenance of any kind, and the disease certainly, during this time, made no perceptible progress. I had not, however, the full confidence of the parents of this child, and I had certainly no cause to be too confident in my treatment of croup; I therefore readily assented to their proposal of taking another opinion, the result of which was the substitution of very rigorous purging with jalap and calomel for the nauseating process, a change with which the parents were much pleased, from the distress which the constant nausea occasioned. The effect of the purgatives was fully produced; but the disease rapidly increased, and the child died, from suffocation, on the evening of the 25th. On making this case the subject of conversation in the presence of a young gentleman, lately from Edinburgh, he informed me that Dr. J. Hamilton, Professor of Midwifery in that University, recommended frequent doses of calomel; but he could give me no account of the quantity of the doses, or of the frequency with which they were to be repeated. Having myself had the advantage of attending the doctor's class in 1779, I referred to my notes taken at that time, but could find only the following vague expression on the subject of this article—'Calomel has been warmly recommended.'

"Case VI.—On the 16th of January, 1808, I was called to see Frederick Bidwell, of Paternoster-row, Spitalfields, a strong healthy boy of nearly four years. It was quite unnecessary to ask one question in order to determine the nature of his disease. He had, on the 15th, the appearances of having taken cold; but he went to bed without much complaint, passed a quiet night, coughed a good deal during the morning of the 16th; but his mother did not notice the unusual noise till three in the afternoon of that day: at seven she sent to me. The countenance was turgid, the shoulders were greatly elevated, the breathing very laborious, and the sense of strangulation extreme; tongue white, skin hot, pulse 150. I immediately determined to try calomel largely, in consequence of the above suggestion, and because I did not know what better I could do; not, however, without taking advantage of the debilitating treatment also:

also: accordingly, 4 oz. (all that could be obtained) of blood were taken from the arm, a blister applied to the throat. Tinct. digitalis gtt. x. were given every four hours, and a powder containing calomel gr. ij. sacch. alb. gr. i. was directed to be given with the greatest exactness every hour. January 17th, 8 A. M. 24 grs. calomel and 70 drops tinct. digitalis have been taken. The symptoms are as nearly as possible the same, the pulse also of the same frequency, no evacuation by the bowels; continue the remedies. 18th, 8 A.M. has taken 28 grs. more calomel, and 90 gtt. tinct. digitalis: the breathing considerably relieved; the cough, though frequent, much less harsh; pulse 124, tongue white, skin cooler; has had two stools of consistence, and of a dark green colour; continue calomel, discontinue tinct. digitalis. 18th, 9 P. M. has taken 20 more grs. calomel: one stool of same appearance, crouping ceased, cough frequent but loose, pulse 120; no medicine. 19th, 11 A. M. breathing easy; cough frequent, but more mild; he expectorates a sort of thin purulent matter, intermixed with pieces of firmer texture and darker colour; his countenance has lost the turgid appearance, and he seems quite at ease; pulse 108, appetite good, B— bound, capt. haust. purg. 22d, has continued to expectorate the same sort of matter, but the mass is now more uniform, resembling well-digested pus. The result of this case was very satisfactory: it shewed that croup was not, necessarily, a mortal disease; and I attributed the more to the calomel, because less blood was taken by two-thirds than in most of the cases which had proved fatal, and because the disease was to the full as violent as any which I had ever witnessed."

Several other cases follow; and the result of the whole is, that calomel, in very large doses, given early enough in the disease, is the remedy most to be depended on. Some valuable observations follow from the American and French writers.

Art. III.—*Observations on Croup;* by Wm. Gaitskell, Esq. Surgeon.

The inquiries principally attended to in this article are,—1. Whether croup is contagious? 2. Whether it is more frequent in northern or southern countries? The first is answered in the negative, without any reserve, though frequent instances occur of the disease attacking more than one person in the same family. It appears most frequent in northern countries, and in maritime districts. That it is more frequent than formerly, appears ascertained by the bills of mortality, as far as they can be depended upon; and, if we may add our own opinion, we should not scruple to confirm such a report, and strengthen it by a remark we have made, that inflammatory diseases of all kinds have increased in number and violence.

The

The 3d inquiry is, What are the common accidental causes of croup; and what the circumstances which propagate it in one country more than another? The 4th, The relative mortality of the disease? 5th, The general treatment? And 6th, Is there any special treatment for croup?

All these questions are answered with very great attention, from the author's own observations, and the facts he has collected by conversation, epistolary inquiry, and printed authorities. The whole affords a truly valuable and comprehensive dissertation on this formidable disease, conducted by a gentleman, who, from his well-known talents, aided by extensive practice in a crowded neighbourhood, is probably the fittest practitioner that could be selected for the purpose.

Art. IV.—*Account of Three Cases of Extraordinary Periodical Sickness, two of which were cured by Arsenic;* by JOSEPH ADAMS, M.D. &c.

These cases are highly interesting, and show in a very pointed view the effects of this powerful remedy.

Art. V.—*Case in which nearly an Ounce of Sulphuric Acid had been swallowed;* by the same.

The vitriol appears by the account to have been vomited in the presence of Mr. Aldridge, the surgeon in attendance, ten days after it was swallowed. We shall offer only the reflections with which the paper concludes.

"On a review of this case, I am aware that considerable scepticism will arise concerning the length of time which the vitriol remained in the stomach; and, perhaps, concerning the possibility of a delicate female recovering from the violent effects of such an accident. I have, however, related the events as they occurred, with the authority on which each of them rests. I shall now add, that to me they are all satisfactory. The mode of accounting for them must be left to those gentlemen to whom we are so much obliged for their experiments in animal chemistry. From some of these we learn, that the vitriolic or sulphuric acid does not affect living animal matter, and that it coagulates mucus. If I am right in this, the above history may be explicable in the following manner: the vitriol was swallowed early in the morning, before any kind of food or drink had been taken to wipe off or dilute the mucus of the mouth, throat, or œsophagus. When received into the stomach it would coagulate the mucus with which it came into contact, and stimulate the stomach to secrete more. By these means it might have been contained in a number of cysts of coagulated mucus, or in one or two large cysts which might have been thrown up at the time Mr. Aldridge perceived the stain and consequent hole in his coat. At the same time, it can hardly be expected that these cysts would be sufficient entirely to preserve all the acid from contact with the sto-

 mach, mach, which, being denuded of its proper mucus, might be inflamed to a certain degree, and thus secrete a substance consisting partly of blood, which, when thrown from the stomach, has frequently this black appearance.

"This suggestion will, I hope, be considered only as hints for the consideration of those gentlemen who have devoted so much time, and with so much success, to this branch of medical science; and, if any better solution of the difficulty should occur, I shall gladly receive it; at the same time, I should be sorry if the relation should excite so unjust a scepticism as to supersede all reasoning on the subject."

Art. VI.—*A Case of Lusus Naturæ of the Female Organs of Generation;* by Wm. Gaitskell, Esq. Surgeon.

In this subject the vagina ended in a *cul de sac,* instances of which have occurred before. The author's reflections, however, are judicious, and his authorities numerous and well selected.

Art. VII.—*Case of the Vermis Lumbricus perforating the Intestinal Canal and Abdomen;* by J. C. Lettsom, M.D. &c.

An abscess, formed in the integuments of the abdomen, when opened, emitted air and pus. After a few days,

"On removing the poultice, a pointed substance in motion appeared in view. It was taken hold of between the fingers, carefully drawn through the orifice, and proved to be another vermis lumbricus in a living active state, in length about nine inches; small air bubbles occasionally appeared; as well as some fæculent matter; both ceased in a few days. For some weeks afterwards, no fæces were discharged through the wound, but seeds of apples or pears, which she had swallowed with these fruits, occasionally protruded. This ceased before the month of December, the orifice of the abscess had cicatrized, and an accretion of the body was perceptible."

Some practical remarks follow particularly on the use of ol. terebinthinæ in worm cases.

Art. VIII.—*Case of Diseased Action of the Heart, effectually relieved by Blood-letting, and Confinement to a Horizontal Posture;* by H. Clutterbuck, M.D. F.M.S.

The importance of this case, related as it is with all possible brevity, induces us to transcribe the whole in the author's words.

"Mrs. Churcher, thirty-five years of age, wife of a person in the employ of Messrs. Calverts, brewers, applied to me Oct. 20, 1814. She had just walked from her own house, a distance of about half a mile.

"Her general appearance at this time was exceedingly distressing. Her countenance expressed great anxiety. The skin in general was perfectly pallid and ex-sanguine, except on the cheeks and lips,

 which which were of a leaden hue. The tongue was clean and moist, but as colourless nearly as the skin itself. Her extremities were cold; pulse weak and irregular; breathing much oppressed; the face looked full and bloated; the legs were swollen as high as the knees.

"She complained of constant uneasiness in the region of the heart, which was affected with frequent palpitations, sometimes coming on even when lying down in bed, but always upon walking, or any other bodily exertion; and, at these times, the pulse at the wrist becomes irregular. Upon making any unusual exertion, this uneasiness is aggravated to the degree of acute pain, which extends to the back, collar-bones, and middle of the upper arms, particularly the left. The menses, she observed, were regular in their periods, but trifling in quantity, and nearly colourless. She fancied she had hardly any blood in her veins; for she said, that, when she accidentally pricks her finger, so as to draw blood, the fluid that issues scarcely stains linen. Her appearance altogether was that of the most complete state of chlorosis, with not a few of the symptoms of hydrothorax or hydrops pericardii.

"Her appetite was extremely bad: she felt always great uneasiness after eating; and the constricted state of her bowels required a constant employment of purgative medicines. Her general strength was greatly reduced; and this, with the growing severity of the symptoms, seemed to indicate much danger, of which she herself was very apprehensive.

"The symptoms now described had continued for several months, and were gradually increasing. They came on soon after a severe attack of inflammation in the chest, which she suffered about a year ago.

"On visiting her the next morning, Oct. 21, at her own house, in consultation with Mr. Key, surgeon, of Fenchurch-street, we found the violence of the symptoms much abated, from her being in a state of rest, and in bed; and the pulse was tolerably regular.

"The symptoms above described sufficiently indicated an excess of irritability and disordered action in the heart; while their duration, severity, and their having succeeded to an attack of inflammation in the chest, gave reason to apprehend, that the disposition to such irregular action in the heart, was the consequence of some disorganization having taken place.

"The constant and, at times, severe pain the patient suffered, and the gradual increase of the disease, rendered it probable that the diseased and inflammatory action was still going on; and all hope of relief appeared to me to turn entirely upon our being able to check its further progress. No means seemed so likely to effect this desirable object as blood-letting. Notwithstanding, therefore, the debilitated and, seemingly, bloodless state of the patient, it was determined to make a cautious trial of it. Upon the pretext of examining the state of the blood, about five ounces were drawn from the arm, though not without much reluctance on the part of the patient; she bore it, however, without inconvenience. After the blood had coagulated, there was observed a full proportion of *crassamentum*, which was of the ordinary consistence, and somewhat cupped or contracted in form, but without any buff upon the surface of it. There appeared to be either a deficiency of the red particles, or else they wanted their usual florid colour, the *crassamentum* presenting almost a leaden hue.

"The patient experienced evident relief from the loss of blood, and the road to further probable advantage seemed by this to be pointed out. The digitalis was administered in small and frequent doses, as a means of lessening the irritability of the heart; as was the ammonia in small quantities, with the view of exciting a little the action of the stomach, and of determining the circulation to the extreme parts. Aperients were exhibited in such quantities as to ensure several evacuations by stool, daily—the use of plain, easily-digested animal food was allowed, as the appetite might require; all strong drinks were prohibited, as they had always been found to aggravate her sufferings; and, above all things, perfect quiet of body and mind, and a horizontal posture, were enjoined; and, for this purpose she was confined to bed almost entirely for the space of ten weeks.

"The blood-letting was repeated at intervals, and the plan altogether persisted in, with great regularity, for nearly three months, with gradual and continued amendment; and, at the end of this period, her health was perfectly restored. She had lost every uneasy feeling about the chest; the pulse became quite regular, and of its natural strength and fulness; the swelling of the extremities disappeared; her appetite returned, and the bowels acted readily with very little aid from medicine. The menses returned at their regular periods, and, the last time, in a perfect manner, both as to quantity and colour. Her complexion, also, was now as good as at any period of her life.

"She was bled, in the whole, four times from the arm, and once by cupping, from the nape of the neck. From ten to twelve ounces of blood were taken away on each occasion after the first. The time preferred for the purpose was immediately after each imperfect attempt at menstruation. Four or five evacuations by the bowels were procured daily, by small doses of aloetic pills, with which a very small quantity of sulphate of iron was combined.

"I have not mentioned a troublesome beating which she felt in the head, at the time that the heart acted most irregularly; as this symptom appeared in a secondary light only, and it yielded with the other symptoms.

"I have related this case, as it appears to me to establish some useful practical points. It serves to show, that symptoms of an alarming kind, seeming to indicate an organic affection of the heart, (or which, at least, threatened to terminate in disorganization,) are not altogether hopeless; that a case which seemed of all others, from general appearances, and according to general opinion also, to be the most unfit for blood-letting, not only bore this evacuation with impunity, but evidently was effectually relieved by it. And, if so, so, it appears further to be probable, that the employment of remedies of an opposite nature, such as tonics and stimulants, which are generally had recourse to in such cases with a chlorotic character, and with swelled extremities, would not only have proved unavailing, but, in all probability, have aggravated the disease.

"I am disposed to attribute much of the advantage received in this case to the strict confinement of the patient to the horizontal posture for such a length of time. I was induced to insist upon this, from knowing the effect which the erect posture of itself has in quickening the pulse under all circumstances; and, particularly from observing the great distress the patient suffered from every bodily exertion.

"August 1815. I am now, after the lapse of ten months, enabled to state, that the patient has continued to enjoy perfect health."

[The remainder of the papers will be noticed in our next Number.]

---

*Edinburgh Medical and Surgical Journal*, No. LI. July, 1817.

Art. I.—*On the Statistical Pathology of Bristol and of Clifton, Gloucestershire.* By C. Chisholm, M.D. F.R.S. &c. &c. and Senior Physician to the Clifton Dispensary.

"I was induced (says Dr. Chisholm) to contemplate the subject of the following paper, as it relates to facts abstracted from speculation, by the result of four years' observation of the diseases of Clifton, as they passed in review before me at the Clifton Dispensary. The facts which further investigation of the subject brought into view, appeared to me highly important, and almost, if not wholly, conclusive, as to the question whether the infection of typhus is specific or not? In the adduction of facts, I have carefully avoided those of a doubtful nature, and have given preference to those of which I acquired a knowledge from the information of gentlemen connected with, or in the immediate medical management of, large hospital, dispensary, or public school establishments, within the limits of the city of Bristol.

"There is, perhaps, no mode of acquiring a knowledge of what may be called statistical pathology so perfect as a parochial or district dispensary. The objects of such an institution are confined to that description of people who are most exposed to the action of local causes of disease, whilst their mode of life removes them from those of an artificial nature. Luxury, with all its attendant evils, has no place in their domestic establishments—superfluity, of every kind, they are unacquainted with, unless it may, unhappily, be that of distress and poverty—whatever morbid causes proceed from the nature of situation and soil, from the changes of the atmosphere, from imperfect ventilation, from the accumulation of filth, from crowded small small rooms, and from poor and scanty diet, these people are the subjects of; and hence a correct and well digested register of their diseases becomes a faithful record of the pathology of such parish or district."

Nothing can be more just than this general proposition, so far as it relates to the knowledge of a district, of the prevailing diseases, and the general inferences deduceable from the whole. The theatre of our author's present practice is so generally known, and, for the most part, so similar to that of other large towns, and their extensive suburbs, that we need only mention a few local peculiarities connected with the period at which these observations were made.

Respecting climate, we are ready to admit, that, in the higher parts of Clifton, it is, if possible, more inconstant than in most other parts of our island. The westerly winds, which, in many places, are mild, arrive here fresh from the ocean, and have a most unfavourable influence on vegetation, as well as on the human frame. The increased population has been chiefly in the more exposed parts, and confined to those individuals whose circumstances are easy. These, therefore, must be left unnoticed when speaking of typhus. "Such local advantages," continues our author, "would be effectual in excluding a morbid constitution, had the inhabitants themselves contributed their own exertion. But here are the unpleasant features of the picture. Nearly three-fourths of the population of the parish are confined to that part called the Hot-well Road, extending about a mile in length along the western bank of the Avon." This description is continued for some length, and with a detail of every disadvantage. Yet, in the midst of the whole, it is impossible not to remark, that three-fourths of a population of 9000 is less than 6000 which extended for the length of a mile along the banks of a rapidly-flowing river, however narrow the space occupied by their dwellings may be, they can never be considered as destitute of free ventilations. That the effluvia from these houses will often be unpleasant to those who are accustomed to the luxuries of better life, cannot be questioned; but the air cannot stagnate for any length of time, especially when we consider the cheapness of fuel as far as that river extends. Add to this, where there is a disposition to "religious observances, and to the perusal of religious books," there must exist a degree of order which implies cleanliness, at least on the return of each weekly festival. In all this account, therefore, we see no cause for the production of typhus; for we perfectly agree with Dr. Chisholm, that the emanations from putrifactive matter are not the causes of infectious fever. For arguments in proof of of this, we are referred to the Doctor's paper, Edin. Med. and Surg. Journal, vol. vi. p. 389; and, to prove our acquiescence in the same, we refer our readers to our xxivth vol. page 422. We shall hereafter show our opinion of the effects of vegetable and animal putrifaction on the human health; but we do not conceive that they make any necessary part of an inquiry into the origin of typhus fever.— We should beg Dr. Chisholm's pardon, and congratulate ourselves in discovering, that this learned author seems disposed, at least for the present, to divest himself of the term typhus, and to substitute another, in which we most heartily accord with him.

"In such a state of things, (says he,) were we to form our opinion by the usual speculations, founded on imperfect ventilation, on the accumulation of filth, on crowded habitations and rooms, and the squalor of the persons inhabiting places subject to such supposed causes of disease, we should infer that the Hot-well Road, or town, was the very abode, the very centre of infection. It will appear, however, from the appended table of diseases, that, although much disease has existed, the supposed offspring of the circumstances I have stated, typhus, or a fever of infection, has scarce a place. How can this be reconciled with what we hear and read of the ravages of such diseases in many of the larger cities and towns of the United Kingdom? It must be confessed, indeed, that the fluctuation and instability of opinion respecting infection, have been as great as they are unaccountable. We may, however, trace their origin to prescriptive ideas. Filth and infection seem closely allied, seem to bear the same affinity to each other as cause and effect; and hence it results that a belief prevails that the accumulation of filth, &c. must necessarily be the cause of typhus,—a belief handed down, not inquired into, and, consequently, converted into an established fact,—an axiom in physics. Little research is instituted, and mankind continue to think and act on a faith not sanctioned by true philosophical principles."

This subject is continued through several paragraphs; but, as we have never considered putrifaction the cause of infectious fever, it is enough that we have shewn there is no want of ventilation, or even of that change in the condition of the air which a weekly cleanliness must, to a certain degree, produce. That, however, the inhabitants do not enjoy vigorous health, is evident, from their "*squalid* appearance," which, we suppose, is not completely changed by their Sunday ablution and change of dress.

"*Fevers of infection*," it is afterwards remarked, "are more prevalent in manufacturing than in commercial towns." The reason of this will be considered hereafter. At present we shall only stop to remark, that, if such is the case with fevers of infection, we shall hereafter shew that with some other other epidemics the contrary will be found to take place. Nor can we impute this difference to the causes assigned by the author, namely, "that the unnatural state in which the inhabitants of manufacturing towns are placed, may dispose to the generation in their systems of that unknown virus, or poison, called typhous infection; whilst the active bustling, and more natural condition of the inhabitants of commercial towns, preserves a due balance in their systems, by throwing off those secretions, useless in the organization of the human body, and injurious to its health when retained."

It is extremely difficult to follow Dr. Chisholm. The following sentence, however, is so precise, that we may fairly quote it as his fixed opinion. "Indigence and sloth," says he, "are often found in the same person, and, when they are, then the retribution necessarily attached to such an unhappy union may be perceived, and may be felt. Such an union I conceive to be *the parent of typhous infection:* such an union, therefore, it is the business, and ought to be the duty, of the enlightened and humane, and more especially if placed in authority, to prevent by all possible means; for, although it may not always be destructive to the individuals themselves, in whom it originates, it must, and is always so to all who come within the radius of the infection which emanates from it." Here, then, we have the author's decided opinion of the origin of that substance which induces the infectious fever. Let us now attend to his account of the manner in which its influence spreads.

"Thus, then, (continues he,) the first set of the first class of causes is the effluvia emanating directly from human bodies infected with contagious or pestilential diseases, or from substances to which the basis of these effluvia has attached itself; *the second proceeds from human effluvia arising from healthy persons, but, from the peculiarity of the circumstances in which they are placed, in a state of morbid concentration, and capable of generating a principle similar to that produced by infectious and pestilential effluvia.*"

By this passage it appears, that, when healthy persons are infected with this air, the effluvia arising from them is only capable of generating a similar principle "*from the peculiarity of circumstances under which they are placed.*" All this is different from the contagions, for small-pox and measles produce their effect in every possible situation, and the only security is, not the *circumstances* in which the persons are *placed,* but under which they *exist*; that is, a susceptibility, or a want of susceptibility, which last is only acquired by having previously gone through the disease. We are glad, therefore, to find the author avoiding the word contagion; but we conceive it would have been much better to to have confined himself to *infections*, without the addition of *pestilential*, because, in philosophical language, no two words should be considered synonymous.

But, though this infectious fever can only be conveyed under certain *circumstances*, yet those circumstances, in Dr. C.'s opinion, are no otherwise connected with climate than in modifying the character of the disease.

"A close and discriminate attention to facts and circumstances render it evident that typhus does exist within the tropics, varying only from the same disease in other climates, in as much as a high temperature in the former generates a peculiar modification of its specific nature, and, in many respects, of its symptoms; and that the malignant pestilential, or what has been most improperly called "yellow fever," (an impropriety productive of all the mistakes, all the controversy, all the warmth of discussion, exhibited by the writers on the subject,) was really and truly the typhus of temperate and cold climates, assuming a monstrous, a new, and most destructive form, through the agency of a tropical climate."

Here how much we have to regret the introduction of the word typhus in the above passage: for, if the fever assumes a new *form*, *its character* is new, whatever may be its origin; and not only the nosological character, but the whole *historia morbi*, as given by Cullen, of typhus, is different from this "monstrous new and most destructive form." If we understand Dr. Ch. the error is two-fold, first in calling a fever typhus, which neither answers the description of, nor is to be treated like, the typhus of authors; and, next, in asserting that it cannot be yellow-fever, because its origin is different from the cause which most commonly induces the fever known by that name. For, if climate is sufficient to give a new character to the disease, and that new character so nearly resembles the fevers of the tropics, it would, in our opinion, be at least safer to name the disease according to its symptoms, and the mode of treatment, than according to its origin. We would ask, whence has the confusion concerning yellow-fever arisen but from the similarity of symptoms? whence the error in the treatment but from the identity of names as often as the word typhus is used? The origin of a disease is part of the inquiry into the means of preventing its extension, and, as far as its character and symptoms assist in that most important part of therapeutics, the name is certainly an object of attention! but, if the symptoms of a disease arising from local miasma, and from infectious atmosphere, are similar, so much of the name as depends on a similarity of symptoms is convenient for all the purpose of diagnosis and treatment. The origin is a different inquiry, and ought never to be confounded with the disease itself. It is a maxim as old as Celsus,* that the same cause, applied to different subjects, produces different effects, and that there must be more than one cause for a fever; on which account that which predominates and gives the character to the disease should be considered as the cause. In the present instance, the climate gives the character to the disease, and is the indication for the mode of treatment. Whatever, therefore, the other cause may be, this is the one to be principally considered. Though what we have now said is not necessarily connected with the statistical pathology of Clifton, yet we could not easily pass it over without some animadversion. We shall now follow the author in his first inquiry.

"It will appear, (says he,) by the table of diseases admitted into the Clifton Dispensary, from 1st January, 1813, to 31st December, 1816, that, of 1699 cases of disease, only 16 were typhus, or only 1 in rather more than 106. This fact of itself is an extraordinary one, when the circumstances of the indigent inhabitants are considered, among whom it has been universally supposed peculiarly to prevail. But, when it is further known that these 16 cases occurred in situations and houses sufficiently well aired, open, and clean, and in persons by no means remarkable for sordid habits; whereas, in the various courts and alleys wherein the air is corrupt for want of due ventilation, and wherein also the rooms and persons of the inhabitants were such as to render them highly offensive, not a single case of typhus appeared, at least not one was reported at the Dispensary; the inference seems reasonable, that filth, &c. are not the causes of typhus, but that it proceeds from a specific virus introduced."

It is much to be regretted that practitioners of such standing as Dr. Chisholm should still require to be reminded of certain well-known facts, which, if constantly kept in view, would solve many, if not all, these difficulties. Dr. C. remarks of the inhabitants of the close alleys, that their persons are squalid; by which, we conceive, is meant their countenances unhealthy, or, if we understand him, that, though not ill enough to prevent their customary occupations, they are by no means free from disease. Is not such often the case with prisoners and gaolers, when the prison is not reported as infected with gaol fever? Yet, under such circumstances, have not the court and jury been infected sometimes to a degree so alarming and so general as at once to shew the cause? Let us then only suppose that an inhabitant of the higher and healthier parts of Clifton, less accustomed than Dr. Chisholm to morbid effluvia, should visit some of these houses on the river-side, or receive one of these squalid inhabitants in his every-day dress. Can any

* Vid. Prefacionem.

thing thing be more probable than that the effluvia from such a source should induce fever? The proper inquiry should be, whether fever, from such a cause, spreads in the higher parts of Clifton in the manner it is known to do in ships, in gaols, and in confined neighbourhoods? Is it not necessary, then, that the parties should be placed in a "*peculiarity of circumstances?*" and is it not absolutely necessary that such peculiarity of circumstances should be accurately traced before any inference can be drawn relative to contagion, infection, pestilence, or miasma? We may further remark, that, though those fevers which occurred at Clifton are called typhus, and probably might have the low type which ought to characterise that disease, yet we have no proof that there have been no other causes for such fevers. Is not the despondency which lately pervaded so many classes sufficient to induce fever, and fever of such a description?

Dr. Chisholm proceeds to make some remarks on the various and incorrect manner in which the word typhus is often applied. Of this we have said enough on many former occasions. Some remarks follow on the Bristol Infirmary, the return of sick for three years, and the proportion of fevers called *typhus*, but which Dr. C., with much propriety, conceives arise from miasma of soil more than any of the common causes of infectious fever.

An account follows of St. Peter's Hospital, which, as far as we can judge, is similar to what the London Work-house ought to be, namely, a receptacle for those poor who cannot claim a settlement, or who are not in a condition to be removed to it. From the care taken to preserve order and cleanliness, "infection has never been generated in the house"—an expression which implies, that, with less care, such an event might have occurred. Ever since the docks have been formed, this hospital has been more subject to sickness. This is very reasonably imputed to the want of that drainage which the building formerly received from the flow of the tide instead of the stagnant water.

"The disease observed as more particularly produced by the exhalations from the almost stagnant water of the river, thus become a floating harbour, is a low fever, partaking much of the nature of synochus, but marked with indistinct intermissions. The number of cases of typhous or infectious fever has been 50, during the period from 1st January, 1813, to 31st December, 1816; and of these sixteen have died, or one in rather more than three, a proportion far exceeding what has happened at the Bristol Infirmary, during the same period, and which seems only to be accounted for by the symptoms of the disease acquiring more violence from the insalubrity of the situation for some years past, and by the condition

 of of many of the subjects when brought into the house. The result, however, furnishes an additional and satisfactory proof, that the virus of typhous infection is specific; for no establishment of the kind can be kept in a more perfect state of cleanliness and ventilation, and, in every instance, the infection appears to have been traced to a source foreign from the house itself. Since the beginning of the present year, *i. e.* from 1st January to 12th March, 1817, about twenty cases of typhus have occurred. The infection was introduced by a wretched coloured man, found destitute in the street, and received into the house, without the nature of the disease he laboured under being ascertained."

The reader will regret with us, that the fevers and their causes are here too much confounded. However, it would appear, that by great cleanliness the infectious fever, when introduced, may be prevented from spreading, and that it becomes more fatal and more general by the accumulation of filth. We are not now offering our own opinion, but what we conceive the fair inference deduceable from Dr. Chisholm's statement; for we are very far from being satisfied with the confused manner in which the first string of inferences is drawn.

"The foregoing facts tend to establish only one partition of the proposition, viz. that typhous infection is not the offspring of *filth, crowded unventilated* rooms, &c.—it remains to adduce facts which go to the establishment of the other partition, viz. that typhus infection does exist, and may be propagated in places the most clean, most freely ventilated, and in all respects the best regulated. If such can be adduced, then the proposition, I apprehend, may be deemed proved, that typhus infection is specific, and not the produce of adventitious circumstances. In this last respect, Bristol is by no means exempted from infection: but, of many instances which could be given, I shall select the following from among public schools, in a charitable foundation, as it seems to place the subject in a particularly clear light."

Now, in our opinion, *filth* should not be confounded with *want of ventilation*, as we find it in the beginning of this proposition. Next, we would ask, if *typhous infection* may be propagated in clean and well ventilated places, what is meant by the word *propagation?* If only that it may be communicated, we have already admitted as much. But may not its further propagation be arrested by cleanliness and free ventilation, and has not the establishment of St. Peter's been brought as a proof of this? May we not further ask, is there any kind of cleanliness or ventilation that will prevent the propagation of small-pox? On the contrary, does it not extend with more certainty in the pure air of a village than in the corrupted air of a large town.

If we understand Dr. Chisholm, no want of ventilation, no no filth, are sufficient to generate that condition of the atmosphere which produces infectious fever. In answer to this, we are ready to admit two things, first, that fever is most commonly introduced into such places, and, even when we are ignorant of such an introduction, it may have existed. But it is certain, that fever from such a cause spreads only in crowded and ill-ventilated communities; and, also, from the frequency with which it is found in camps, fleets, and prisons, and from the circumstances under which it occurs in such places, there is every reason to believe that it is generated by such circumstances. Justice to Dr. C obliges us to insert the whole of the following long paragraph.

"Another source of error, or rather of misconception, with respect to the existence of typhus infection among the indigent inhabitants of Bristol, is the occasional occurrence of scarcity of provisions, almost amounting to famine, and the substitution of corrupted or not sufficiently nutritious articles of food. I engaged pretty largely in the discussion of this interesting subject on a former occasion (see Edinburgh Medical and Surgical Journal, vol. vi. p. 412-415); and I have reason to believe the conclusions I there drew are correct and satisfactory. Several instances are on record of epidemics being the consequence of scarcity of provisions and deteriorated food in Bristol; but, from what has been stated in the work referred to, and from the imperfect information I could obtain of the nature of those epidemics, I am induced to relinquish every idea of their originating in infection, although the usual, indeed general, opinion entertained is, that they were infectious, or typhous fever. In the annals of Bristol, instances are recorded in the years 1597, 1608, 1752, 1765. The most recent happened, I believe, in 1795 and 1799. These were attended with a very fatal epidemic fever. Many afflicting details of the disastrous consequences have been given to me by gentlemen, who, as agents of charitable societies, took a very active part in the investigation of cases of distress, and in their relief. Misery of every description prevailed; but the fever, as far as I could collect from this source of information, was evidently a fever of exhaustion, not of infection. From the medical gentlemen who at that time practised in Bristol, I could obtain no precise account of this direful calamity,—a deficiency much to be regretted. That famine may sometimes be the precursor of pestilence, I believe; but it is not so from the mere privation or deterioration of food; it is from the superinduction of infection, to which, under such distressful circumstances, the poor are more exposed. Riverius has observed, 'Quando magna adest annonæ caritas et penuria, unde vulgare illud, ὁ λοιμος μετ' λιμον pestis post famen.' It is a vulgar observation, but it is also a vulgar error. A disease, however, equally fatal, does arise from the exhaustion consequent upon famine; and, the symptoms of it assuming the features, in some degree, of typhus, it is thence too frequently mistaken for it, to the irremediable loss of the unfortunate sufferer. This disease is the

Asthenia abstinentium of Sauvages (Nos. Meth. tom. i. 805)— ‘Cutis árida, flava, rugosa, os arescebat, lingua et dentes nigrescebant, vox raucescebat, macies magna, nulla perspiratio, dejectio, mictio, *&c.*’—a disease which in Scripture is designated by two words, מזי רעב, *rob mui, fame combusti*, which in the English version is translated ‘they shall be burnt with hunger,’—(Deutr. xxxii. 24.) This is the denunciation of the Almighty himself, and is as awful as it is grand. The expression has been variously rendered, according to the conception of the effect of famine formed by different nations. Thus in the Septuagint it is τηκομενοι λιμῷ, liquescentes fame; the Latin version of the Syriac has it *conturbabuntur fame;* of the Chaldaic, *inflati erant fame;* but the original in one word comprehends the whole. Although rather foreign to the object of this paper, I cannot forbear giving myself the gratification of directing the attention of the reader to a valuable paper of Mr. Bacot's, in the seventh volume of the Transactions of the Medico-Chirurgical Society of London, in which a most instructive description is given of this species of asthenia, as it fatally prevailed in a battalion of the guards serving in Spain in 1812-3. I the more readily insert it here, because it is a disease that I believe has never been accurately described before. ‘The patients usually came to the hospital complaining of chilliness, languor, and depression, both of strength and spirits; the countenance wan and melancholy; the pulse small, frequent, and tremulous; and the surface of the body unusually cold to the touch. Giddiness of the head was a frequent complaint, and a deep and constant sighing was an universal symptom; yet there were none of the common attendants of the first attack of fever, no violent headach, nausea, or thirst, no accession of heat, or marked rigors, in the first instance. I have seen numbers of men brought to the hospital so attacked die in twenty-four or thirty-six hours after their admission, without a prominent symptom, insensible to every kind of stimulus, and never having any accession of heat or increased action of the vascular system, from the moment of the attack to the hour of their death. In many men of very robust habit, the disease assumed more of the common forms of fever, and very soon put on the typhoid character, with parched tongue, low muttering delirium, and terminating, in some instances, in a suffusion of bile over the whole surface of the body.’ (p. 379.) This is the disease so often among the poor mistaken for typhus, because frequently whole families are prostrate under its direful influence. Many instances of this occur every winter, but at the periods stated more remarkably, because the cause more extensively and more severely existed. The present year would have pre-eminently furnished elucidations of this fact, had not the consequences of famine been in a great degree averted by the extremely judicious measures adopted by the mayor, and cheerfully acceded to by the more wealthy inhabitants. It may not be irrelevant to remark, that the misapplication of the word typhus, so frequently occurring in this lamentable species of asthenia, may serve, perhaps, as a guide in detecting detecting similar misapplication in those extraordinary accumulations of fever inserted in the reports of fever under the denomination of typhus, which we hear and read of in other towns and cities equally populous as Bristol, and equally exposed to the causes of asthenia abstinentium."

We confess some parts of this are so much like what is usually called typhous fever, that we know not how to make the distinction. We prefer the word Camp Fever, and leave our readers to the description, as they will find it in various writers, particularly in Dr. R. Jackson, whose name we have so frequently introduced, and our remarks on whose works occur in so many of our volumes.

The remainder of the paper consists of very long and learned quotations and authorities concerning the periods at which epidemics have visited Bristol and other places. After which, follow a table of the diseases relieved at the Clifton Dispensary for three years. The number of patients amounts to 1699, of whom died 101, or about one in seventeen. Among the fatal cases, phthysis makes twenty-three; of rubeola, five in fifty-five, a large proportion in a disease generally considered mild; of typhus, sixteen, of whom one-quarter died. We were struck with some of the names: scrofula *vulgaris*, fifty-four, of which two died. What is scrofula, and what are the other distinctions? Elephantiasis, on the other hand, has no specific or trivial name, yet the various senses in which authors use that word are repeatedly noticed.

Such are the general outlines of this long paper. We know not in what terms to speak of it, and trust the ambiguity of some parts will be a sufficient apology for our requesting Dr. Chisholm to revise it, giving a precise meaning to each of his terms. This we particularly request, as it may furnish us with many useful hints when we venture to accomplish our promised dissertation on Contagions. At present we shall only in general remark, that we wish to confine the word contagion to diseases, which, under all temperatures and in every known state of the atmosphere, spread from a diseased subject to all who are susceptible of the same, and which can originate as far as our knowledge now extends from no other cause; that marshes produce intermittents or remittents, in proportion as their myasma is more concentrated; that filth has an effect on certain pestilential diseases; and that camp, hospital, or gaol, fever may be generated wherever sick of any description are crowded.

An Appendix, by Mr. Cumberland, gives the geology of Bristol and Clifton, according to their various strata.

Art.

Art. VII.—*Extract of a Letter from Mr. George Birnie, Assistant-Surgeon of his Majesty's Ship Antelope, dated St. Christopher's, 4th January* 1817, *containing Observations on Yellow-Fever, to James Robinson Scott, F.R.S. Surgeon, Royal Navy, &c.*

This paper contains so much matter connected with Dr. Chisholm's, that we have been induced to insert it here, though not in order. It is a very useful paper, and shows industry, genius, and many very good qualities. It is not, however, without very considerable attendant faults. These we excuse in the writer on account of certain passages which, in justice to him, we shall transcribe in his own words. They are contained in the introductory and concluding parts of his letter.

"I now set myself down to give you a brief statement of the observations which I have made respecting the fever of the West Indies. We have suffered severely by it; and I have to lament the loss of two of my dearest friends, who fell early victims to its indiscriminating malignancy. My opinions concerning its origin, nature, and cure, differ in some degree from those of all the other medical men on the station; I am, therefore, induced to lay before you the result of my experience, for the benefit of your friendly inspection; and shall expect your observations thereon with the greatest anxiety."

The writer concludes.

"I have now given you the sketch which I promised, as far as *I observed the nature and course of this fever in the Antelope and Childers*, and I beg that as soon as possible you will write to me, and let me know in what we agree and disagree on this subject. If you think this letter worth inserting in any of the medical journals, you are at liberty to do so, or as you please with it. When in the Childers, I had a most severe attack of this fever, and was *given over* by the physicians at Barbadoes Hospital for five days, but I recovered."

By these passages it is evident, that Mr. Birnie, who is an *assistant*-surgeon, conscious of his own want of experience, has given only *what he observed in the Antelope and the Childers*, waiting with much anxiety for his friend's opinion, and committing the fate of his letter to the same friend. After this, we are obliged to address our animadversions to Mr. Scott, a *full*-surgeon in the royal navy. For the same reason, we shall be as sparing as possible of remarks, and copious in extracts, considering that, in a production like the present, the facts related are all that the writer is answerable for.—The writer continues,

"Like every other non-tangible subject of importance, the fevers of the West Indies have engendered a vast variety of opinions. The

The utility and beauty of classification have given way to the rage of discovery; and every one comes forward with his little bit of an hypothesis to set the world to rights, and claim the rewards of genius. Had I not matter of greater moment to communicate, I could give you great amusement by the recital of many an absurd explanation of the nature and causes of yellow-fever. I shall here attempt to disprove all the great positions of Mr. Pym. His own words, the authorities he quotes, and the result of my own experience, have led me to a very different conclusion from that which he has adopted; but, as I am a young man, and a very young practitioner, as my opportunities for observation have been few, and perhaps my attention slight or ill-directed, it would ill become me to be dogmatical. I shall, however, with a faithful, though feeble, hand, trace what *I have seen* of these diseases, endeavour to prove their individuality, and that they are generated in the West Indies, without a possibility of supposing their origin or increase to have been at all connected with contagion.

"Mr. Pym is the first, *as far as I know*, who has attempted to prove that the fevers of the West Indies differ with regard to their nature and origin. He divides them into three kinds, the continued bilious, the remittent bilious, and the Bulam or yellow fever. His diagnostics are, that, in the first, as the disease advances, the skin becomes of a *very deep yellow colour;* in the second, of a *deep yellow;* and in the third, of a *pale orange colour*, with the addition of a peculiar drunken appearance of the eyes. He says, that, in the first, there is never the black vomit; in the second, seldom, if ever; and in the third, always. The mere statement of these definitions demonstrate indubitably that the diseases in question are but grades of the same affection. Here the black vomit is the only characteristic symptom of Bulam fever (for the drunken appearance of the eyes takes place in fevers of every different type;) and he even allows the black vomit to appear sometimes in the remittent, though he denies it ever to take place in the continued bilious. It is a symptom universally looked on as the immediate precursor of death, and cannot be called a diagnostic. This is a brief statement of Mr. Pym's doctrine; and I shall now proceed to lay before you as short and clear a view as I am able of the forms under which I have seen the endemics of this country, by which you will be enabled to judge of the argument and its utility for yourself.

"Immediately after our arrival in this country, about the beginning of March last, when the inhabitants of Bridgetown [Barbadoes] were perfectly healthy, and no cases of fever on shore, at least I am certain that no one belonging to the ship had been near, or indeed had heard of any, sick person on shore, a fever, characterized by all the symptoms which Mr. Pym has attributed to Bulam fever, made its appearance on board the Antelope; and, since that period, 110 cases have occurred in her, of which thirty-one only have died; of those thirty-one, nine either lived entirely in the fore and after-cockpits, or messed, and, consequently, passed the greater part of their time, there. None who had black vomit recovered; and of the thirty-one, seven only had black vomit; and of these seven, six were of the nine mentioned above as living almost entirely *below*, where the atmosphere in this country is thick and heavy, and produces a peculiar hot sensation on descending from above into it. The temperature is always about the same, is often below what it is on deck, and, from the continual burning of candles, the crowding together of several people, the debris of pantries and mess-rooms, not always exceedingly clean, together with the want of circulation of air, may have caused so great a proportional number of those who were obliged to mess or live below to die, and have black vomit. If we compare the mortality amongst them to that of the rest of the ship's company, I conceive the observation will be of infinite importance in a future stage of this investigation. The patients at first universally complained of a pain extending across the forehead, confined to a line above the eye-brows; frequently the eyes could not bear the light, and pain was always produced by slightly pressing on the eye-balls; the pulse was sometimes natural, at others full, quick, or interrupted; the skin always hot, dry, and pungent; *irritability of stomach* came on on the first, second, or third days; the matter rejected from the stomach was indigested food or drink, bilious matter, and, in several, towards the latter stage, that peculiar secretion called black vomit. We have lost fourteen officers and seventeen men by this disease, and it possessed, in an eminent degree, *all the symptoms* attributed to Bulam fever, particularly black vomit. Its course was from two to ten days, but generally terminated in from three to four. This is a concise account of the disease, as it appeared in the Antelope, and it was *called Bulam fever*.

"In August last a disease broke out on board the Childers brig, while anchored in the Gulf of Paria off Port Spain, Trinidad, where the squadron had gone to pass the hurricane months. As her surgeon and assistant were both attacked, I was sent to assist Mr. Brown of the Scamander in taking care of the sick. At that time, twenty-eight out of ninety men were labouring under a disease which made its appearance by the same circumscribed pain of the forehead, affection of the eyes, variable pulse, hot, dry, pungent skin, and succeeding irritability of stomach, which ushered in the disease on board the Antelope.

"In the first ten days about thirty persons died: three women and two infants fell victims to it; and this was called *bilious remittent*. But the only difference which to me appeared to exist between the disease on board the Antelope and that on board the Childers was, that, in the former, black vomit appeared in seven cases out of 110, while on board the Childers I observed it in one case only. It was, indeed, said to have existed in the cases of two of the women, but, as I was seized with the disease myself before they died, I cannot assert that they had it on my own authority, and that the disease on board the Childers was attended with a much greater mortality. But, premising that the same causes do not always produce precisely the same effects on the human body, this this difference of termination may, perhaps, receive some elucidations from considering the following circumstances.

"The disease appeared in the Antelope immediately on her arrival in the West Indies. The Childers had been nearly six months in the country when the disease broke out in her. The Antelope was in good order, clean, and well aired, with the exception of the cock-pits, as already mentioned: the Childers was the opposite of all these. On board the Antelope twenty-five died who had no black vomit; on board the Childers one died who had black vomit. Can it be supposed, that the twenty-five who died in the Antelope, without having black vomit, were different from those who exhibited similar symptoms and died about the same time, but who had black vomit? I think not. When the *peculiarly circumscribed pain of the forehead,* and the great and almost unconquerable irritability of stomach, were the distinguishing and leading symptoms in every case; because a certain appearance takes place in a limited number, shall we call them by different names? Let us pursue the comparison still farther, and we shall find that the mortality and liability to attack was equal among those who were obliged to live or mess below in the Antelope, and those who lived on the lower deck of the Childers. The lower deck of the Antelope, where the people mess, is always well aired, except in a heavy sea, which does not often occur in the West Indies, and is always kept in an exceedingly clean state, which accounts for the disease not running through the ship's company, and for its comparative manageableness; but, in the cock-pits, every person was attacked, except two seasoned hands: about one half died, while, in the other parts of the ship, not more than a fourteenth, viz. nine of the thirty-one who died on board her had either lived entirely or messed in the cock-pits, and, of the seven cases of black vomit which occurred on board, six were of those nine. The lower deck of the Childers, where the people mess, was dirty in the extreme. On lifting the hatches of the fore or after holds, a horrid suffocating stench issued from them: it was confined, lumbered with lockers, and the heat increased by the fire-place being on it. It was in a nasty filthy condition, and I do not recollect that it was attempted to ventilate it by wind-sails. This state of the vessel, the want of accommodation and attendance, and the sudden fall of the medical officers, accounts for the mortality on board of her, and the disease attacking all her crew:—only three escaped an attack, and about half died. Here we observe a remarkable coincidence between the extent and mortality of the disease in our cock-pits and in the Childers. We see the influence of the climate modified by the different situation of the two ships, and by the different situation of the parts of the same ship.

"From having observed that this influence is the leading character of these diseases; that, in every individual case of them, the first symptom was *the circumscribed pain of the forehead,* pain of the eyes, particularly on pressure; that all cases were alike characterised by irritability of stomach and rejection of great quantities of bilious matter; that six of the cases in which black vomit occurred were placed in peculiar circumstances; that the other was in a bilious irritable habit, and took place immediately on our arrival; that, of the thirty-one who died, nine were obliged to be almost entirely below: I say, from having observed all the essential and cognizable symptoms to be the same in *both* these diseases (though I cannot point out exactly what combination of circumstances, or modification of causes, produced so great a number of cases in our cock-pits, in which death was preceded by black vomit, and why a still more nasty state of the lower deck of the Childers so few), I am of opinion that they are essentially the same, and fear that medical observers, in the too eager pursuit of *discovery*, have been inclined to look over the thousand phenomena which establish their identity, and to find out and magnify the irregularities which occur in this, as in every other disease. For my own part, I grieve to see so much patient inquiry, and intelligent observation, frittered away in the futile attempt to convert classes into species, or in the insignificant invention of a new name for an old disease. As they all agree that the practice in *both* must be the same,—that this difference, if ever so well established, would lead to no practical result,—why so eagerly maintain an hypothesis, with only one useless dubious argument or fact to sustain it, and which is opposed by every character of identity and principle of classification?"

"The Tigris and Scamander frigates have also been visited by this dreadful scourge, but have not lost more than five or six persons each, which exemption they owe to their high state of discipline, their cleanliness and attention to ventilation; while the Brazen, under circumstances somewhat similar to those of the Childers, has lost *twenty-four* out of eighty men. I do not mean to say that she was dirty, or in *bad* discipline; but her lower deck, where the people live, is narrow, crowded, and has neither ports nor scuttles, with two decks above it. The different degrees of mortality exhibited in the different ships of this little squadron, according to their different degrees of *description*, *ventilation*, and *cleanliness*, I think sufficiently proves that all the various appearances of these diseases arise from the same origin, and are effects of the same cause, modified, as we have seen, by various circumstances.

"Dr. Ferguson, Inspector of Hospitals, whose experience of the climate and fevers of the West Indies has been very extensive, and entitle him to the greatest deference, has, with much kindness and condescension, communicated to me some facts respecting the attack of this malady, as affected by *local circumstances*. He says, 'that on the tops of the mountains, and in all elevated situations in the West Indies, the only fever known to exist is the ague or intermittent of Europe. As you descend, you find the remittent in a mild form; still lower it appears in an aggravated type; until at last, on a level with the sea, in the crowded ill-ventilated towns, which, for commercial reasons, are almost all situated to leeward, over *the beds of dried-up rivers, or swamps*, it appears with all its dreadful and destructive malignancy, rapid in its course, and accompanied with *black vomit*. It has also been observed, that, in very dry seasons, the the *ague* appears lower; and that, in very wet ones, the remittent occurs in *higher* ones.' This gradation will account for these endemics prevailing in different situations according to the season; and I conceive it to be perfectly conclusive in proving their identity. Speaking of what *he calls* Bulam fever, Mr. Pym says, 'it is a disease, *sui generis*, of foreign origin, contagious, and attacking a person once only during life.' That it is the same as the bilious remittents, I think I have made sufficiently evident. With respect to its being of foreign origin, I ask, Do the same causes exist no-where for its production but in Bulam? When it has disappeared for many years, or for months, as it did in the Antelope, where did it hide itself; and, being so fatal, how was it concealed and retained in existence? I consider the doctrine of contagion to be altogether unfounded, and eminently mischievous; and that none of these fevers, allowing a difference, is contagious, I think I may assert on the most solid proofs the subject admits of. On board the Antelope, no precaution was taken to prevent contagion, yet, out of 320 men, having the freest intercourse, not more than 14 or 15 at a time were attacked. The surgeon, two assistants, and surgery-man, were always among the sick, and the three last slept continually in the midst of them, and none of them had the slightest attack of fever, *for many months afterwards*, and only the two assistant surgeons, after being sent, one into the Childers, and the other into the Brazen. The officers frequently came to see the sick; their messmates were allowed to visit, wash, and dress them; yet no appearance of the fever having been ever communicated by contagion occurred. On his being attacked, I lent a pillow to one of my messmates, who died eight days afterwards, having had *black vomit*, yet I slept on it for four months without any other precaution than that of *changing its case*, and without the least attack of fever during all that period. On board the Childers, where they had the fever, which my medical superiors called remittent, out of ninety men, only three escaped being attacked, and every person impelled by duty to remain for any length of time on board was seized with the same disease. The surgeon of the Brazen sloop had been left behind, and on the brig's arrival (for she had been sent from Trinidad, that her men might be conveyed to the hospital at Barbadoes,) he nobly volunteered his services, and fell a victim to his humanity. Another surgeon and three assistants sent on duty into her, had very severe attacks; and three clerks, made pursers into her, died in less than as many weeks. This *appeared* like contagion, but *was not*, for none of the physicians, surgeons, assistant-surgeons, attendants, or nurses, who were continually with the men and officers after their being sent to the hospitals, were ever in the slightest degree affected with any thing like this disease. That the immediate and exciting cause existed on board the brig, is evident from this relation, and from the fact, that her men were taken ill every day, until she was unloaded and cleaned out, and that the black Creoles employed in this service were also seized with the disease in a day or two *after being on board*, but that since this purgation she has

 had had no new case of fever, and but one of relapse. I noticed above, that one third of those who died on board the Antelope, either messed below, or lived there entirely, where they were, in some degree, under similar circumstances with the crew of the Childers; and I have no doubt, had those strangers who were attacked in the Childers been sent into our hold, steward-room, or cock-pits, to breathe a close vitiated atmosphere, loaded with every miasm which such a state is calculated to produce, that they would have experienced some similar attack; or, had our ship's company been crowded down there, so many would not have escaped an attack, nor so many who were attacked have recovered. I think sufficient evidence is here given to prove that *these* fevers are not at all contagious. The fever in the Childers was endemic, not contagious; and it is by confounding the different ideas conveyed by these words, that many wander in a labyrinth of their own construction. This disease is *in all cases endemic;* it is the result of the influence of climate, modified by all the circumstances which I have already mentioned, and perhaps many others; and it is the different nature of these modifying circumstances that determine its nature and appearances. There is no such thing as idiopathic fever. Every effect must have a cause; and no cause can act in the general system, but must affect it through an organ or set of organs. Disease, as well as motion, must originate from a single point; and the power that affects that single point generates the disease, and the climate is that power. With respect to the statement that the disease attacks a person once only during life, if the evidence I have adduced to prove the identity of these diseases, and that they are not contagious be conclusive, this position falls of course; and it is well known that the remittents of this country attack the same person frequently, though each succeeding attack is generally milder, in proportion as the constitution becomes *creolised.*"

After so copious and uninterrupted an extract, we may be allowed, without the danger of any imputation on our candour, to call the reader's attention to certain passages. 1st. That the Bulam and yellow fever are the same. 2d. That, on board the Antelope, the fever originated in the ship, and the mortality was greater in proportion as the men lived below in atmosphere confined, though often cooler than on deck. 3dly. The mortality in the lower deck of the Childers was very similar to that of the cock-pit in the Antelope. 4thly. Bridgetown was healthy when the Antelope arrived—we ought to have been informed of the health of the squadron in the Gulph of Paria, where the Childers anchored in August. But, 5thly, The lower deck of the Childers was dirty and ill-ventilated, and the mortality great in proportion.

Let us now remark, that the crew of the Antelope, though always clean, was seized as soon as she arrived in the West Indies. The Childers, though dirty, remained free for six months months after its arrival. The periods of the Tigris, Scamander, and Brazen, are not marked ; but it is observed in all that the degree of disease was in proportion to their want of ventilation and cleanliness. Mr. Birnie and his fellow-assistant, who escaped on board the Antelope, were seized, one on board the Childers, the other on board the Brazen, both in a similar condition. On board the Childers, even the Creole blacks taken from shore were seized ; but the crew sent to the hospital at Barbadoes did not infect any one, and the ship, when well cleaned and ventilated, was found equally inocuous. From all this, Mr. Birnie concludes, that the disease was endemic, but not contagious; that is, we presume, that it originated with the *people*. But, says Dr. Ferguson, the type of the disease depends on *local circumstances*, and is different on mountains, in vallies, or swamps, or the beds of old rivers. This, therefore, refers to places, and is rather επιχωριος than endemic.

From the above, we may fairly conclude, as we ventured to hint in the preceding article, that confinement and want of ventilation will generate a ship fever in the West Indies as well as in England, though its form may depend on climate and other causes. Hence, in the West Indies, the influence of vitiated atmosphere, whether from confinement or swamps, may produce fever with a yellow skin. In other words, that a ship fever may originate between the tropics, and be conveyed, in a similar form, from Bulam to the West Indies; or, if it originate in Europe, it may be conveyed to the tropics and assume a new form. That such an atmosphere may, like that of gaols or poor-houses, prove even more deleterious to strangers than to those among whom it originated, but that such strangers cannot propagate the disease, excepting in a confined atmosphere. Hence, that the contagion, if any exists, is different from that of small-pox; that it is also different from the miasma of marshes, but that, in the latter, the effects, particularly the yellowness of the skin, may be similar. That hence a yellow fever may be excited between the tropics either by the vitiated atmosphere of a ship or by the miasma of a swamp, and that the mode in which each spreads will depend on the exciting cause, though the symptoms of the fever may often be the same. That, therefore, a fever between the tropics, excited by any cause excepting the exanthemata, may come under the denomination of yellow fever, as far as relates to symptoms and mode of treatment. We are, however, by no means partial to the term yellow-fever, but we think it less likely to mislead the learner than *typhus icterodes*, because the ship fever in the West Indies, in its character and its mode of treatment, ment, has a nearer resemblance to the fever from miasma in the same country than to the gaol or poor-house fever of England; and because, in all acute cases, we conceive the symptoms, and not the cause, of a disease, are to direct us in the immediate application of remedies.

Art. II.—*Some Observations relative to the new Method of Extracting the Cataract, as proposed by Dr. Löbenstein Löbel, &c.* By Edward Chapman, Surgeon, Bath.

We shall take an early opportunity of repeating the author's experiments, before we offer our opinion on this article.

Art. III.—*Contributions to Diagnosis.* By Marshall Hall, M.D. &c.

These relate to Hydrothorax.

Art. IV.—*Case of Morbus Cœruleus arising from Ulceration of the Lungs, and accompanied with the Medullary Sarcoma of Mr. Abernethy.* By William Howison, M.D. Member of the Royal Medical Society.

This case, though by no means uncommon, is extremely interesting. We have several times expressed our wish that, in the treatment of internal tumours, our brethren would show greater courage; and, after the bold attempts lately made in obliterating large arteries seated within the peritoneal cavity, we may expect that in diseases as necessarily fatal as aneurism, similar attempts will be made. Our meaning will be better understood by giving the leading particulars of the case.

The following are the external indications of the tumour during life.

"Situated on the outer edge of the sternal origin of the left sterno-mastoid muscle, there is a large, hard, incompressible, somewhat irregular, and moveable tumour, extending hence along the upper margin of the clavicle, backwards nearly to the trapezius muscle, upwards to the angle of the lower jaw, and reaching anteriorly to the trachea; its basis seems to be very extensive, and deep-seated; its investing integuments are of their natural appearance; and its presence is reported to give no impediment to the performance of respiration or of deglutition. Pulsations, synchronous with the pulse at the wrist, but obscure, are to be felt in the swelling; and the belly of the sterno-mastoid muscle seems to be involved in it."

The patient suffered much for a few days with pain and dyspnœa, and died in the hospital.

"*Sectio cadaveris.*—The integuments covering the thorax being divided, the fat was found to be of a firm consistence, and to extend tend about an inch and a half in depth. The sternum being cut from the ribs, and laid back, a considerable quantity of air escaped with violence from the right cavity of the thorax. The right lung was compressed to the size of a person's hand, and, upon examining its external surface, an ulcerated hole, about the size of a shilling, was discovered, near to its centre, through which the air had escaped into the cavity of the thorax, and compressed the lung. The internal substance of the lung, when cut into, presented a tuberculated appearance throughout its whole substance. The left lung was also found to be much compressed, and smaller than natural; and its internal structure presented the same tuberculated appearance, although in a much slighter degree.

"The pericardium, containing the heart, was found forced entirely into the left cavity of the thorax, and was pressing upon the left lung, so as to impede, in a considerable degree, its free action. Upon laying open its cavity, there flowed from it about ℥vi. of a transparent yellowish-coloured fluid. The heart was larger than natural, much loaded with fat, and its coronary vessels were distended with blood. The right auricle was turgid with blood, partly coagulated, and partly fluid; the foramen ovale was found completely closed; both ventricles were also filled with blood; every other part of the heart, valves, &c. were found to be sound and healthy. The contents of the thorax being removed, its internal cavity appeared to be large and roomy.

"From want of time previous to the funeral, the cavities of the cranium and abdomen were not opened.

"*Sectio tumoris.*—Upon dissecting back the surrounding integuments from the tumour, the belly of the sterno-mastoid muscle appeared greatly diminished in bulk, and the muscular fibres immediately over the tumour were almost absorbed. The sterno-hyoideus muscle was found to be in a similar condition. The superficial veins in the neighbourhood were considerably enlarged, and distended with blood. The sterno-mastoid muscle being separated from its attachment with the sternum and clavicle, and reflected upwards, the tumour appeared to consist of three masses, distinct from each other, excepting at one side, where they were slightly connected. Underneath, and stretching into the axilla, a number of enlarged glands, of different sizes, could be detected by the fingers. After injecting the left common carotid artery, at its origin from the aorta, with vermilion injection, its diameter was found to be considerably enlarged, but its trunk was entirely unconnected with the tumour, excepting by means of these branches which supplied it with blood, and which were considerably enlarged, cellular substance every where intervening betwixt it and the tumour.

"Upon dividing the largest tumour into two equal halves with a clean scalpel, it was found to possess all the characters of the medullary sarcoma of Mr. Abernethy.

"Upon inquiring into the previous history of the above-mentioned patient, we learned that she had led a very laborious and active life, from her earliest years, as a farm-servant in the country,

 and and that she had enjoyed perfectly good health, until within these last two years, when the above-mentioned symptoms commenced, and gradually continued to get worse. The tumour in her neck commenced six years ago, and, when first observed, was about the size of a horse-bean, and, since that period, it has gradually continued to enlarge, but particularly within these last two years.

"The cause of the livid colour of the integuments in this patient (or morbus cæruleus) appears to me to have been owing to the very imperfect circulation of blood through the lungs, and, consequently, to its having been very imperfectly supplied with oxygene from the external air during respiration. The right lung, as appeared upon dissection, was compressed to the size of a person's hand, by the quantity of air which had escaped from the ulcerated hole in its surface, and must, consequently, have been rendered almost incapable of performing its natural functions. The pressure of the air from the right cavity of the thorax upon the mediastinum, during the action of the right lung, had also forced the pericardium and heart totally into the left cavity of the thorax, and, of course, greatly diminished the functions and size of the left lung. The diseased tuberculated state of the substance of both lungs themselves must also have contributed greatly to produce the disease. The action of the heart itself and arteries, upon examination before death, appeared to be irregular, stronger, and fuller at one time than at another, which might arise from the pressure retarding their free action. The pressure of the tumour upon the trunk of the left internal jugular vein, particularly when the patient was lying upon her right side in bed, might have produced the great turgescence of blood in the face, and head-ach.

"With regard to the treatment of the tumour, in a surgical point of view, there must have been much hesitation and doubt, from the uncertainty with regard to its nature, as by some people it was supposed to be an aneurism, and its connexion with the surrounding parts, which were of such importance to the animal economy. From its immense size,—from the important situation which it occupied, imbedded among the large blood-vessels, nerves, glands, supplying the head and neck,—from the uncertainty whether or not the common carotid artery might be immersed in its substance,—from its depth, the serious nature of the operation, and the debilitated state of the patient's constitution,—its extirpation was delayed, and she was recommended to the physicians, under whose care she eventually died. By the dissection, all these circumstances were explained; and it became evident, that the tumour might have been removed without risk, excepting from the extent of the wound, and the debilitated state of the patient's constitution, arising principally from the internal disease under which she at the same time laboured."

In this instance there was much pulmonary disease, besides the distress arising from the tumour; but it not easy to say how much of the whole distress arose from the tumour. We perfectly agree with the author in the danger of cutting out such a substance; but we can see no objection to making an opening opening through the integuments into the substance of such tumour. The contents, as far as is necessary for the immediate relief of the patient, would be pressed out by the act of respiration, and though the part might not heal for many months, yet the patient would have a better chance of recovery.

In our next, we shall notice the other original articles, which are more than usually numerous in the number before us. Though somewhat out of place, we think it right to acknowledge, that, from our eagerness in attending to the important facts in Mr. Birnie's paper, connected with prophylactics, we omitted his method of cure. It is "contained in very few words;" and consists, chiefly, in very free venæsections. His opinion of calomel and bathing, particularly the former, is similar to Dr. R. Jackson's.

---

[As the Editors of the New-England Journal have done us the honour to adopt our review of the Medico-Chirurgical Transactions, we make no apology for availing ourselves of their labours on a work which we have not yet been able to procure, especially as it affords us an opportunity of offering the opinions of others on nosology and contagion.]

*First Lines of the Practice of Physic.* By WILLIAM CULLEN, M.D. late Professor of the Practice of Physic, in the University of Edinburgh, &c. &c. *With Notes and Observations, practical and explanatory; and a preliminary Discourse in Defence of Classical Medicine*, by CHARLES CALDWELL, M.D. 2 vols. 8vo. Philadelphia: Edward and Richard Parker, 1816.

IT is somewhat singular that while Dr. Cullen contributed, perhaps more than any other person, to diffuse among medical men those principles of physiology and of pathology which now prevail, yet his own peculiar theories scarcely find a single defender among the physicians of our day. His works, and that before us more particularly, are highly valued and are diligently read, but not for the sake of the peculiar theories they present to us. The excellence of Dr. Cullen is, that he gives his descriptions and his theories distinctly; and that he is peculiarly clear and just in his delineations of disease. As a systematic writer, he is, perhaps, unrivalled in this respect. In regard to individual diseases, there are, no doubt, many who have given descriptions as faithful, and, of course, more full and minute, than those of Cullen.

But the merits of this distinguished teacher are universally known, and we have not taken up the work before us with an intention to review his writings. Our business is with *this edition* of the "First Lines, &c." in which is found much matter peculiar to itself. This matter consists of a preface and a preliminary discourse, and of numerous notes or commentaries. In these notes, the intention of the editor is, not to explain the doctrines of the original work, but to furnish corrections in respect to theory, and to supply deficiencies in respect to practice. As the editor is a gentleman who ranks very high among the physicians of Philadelphia for his literary and scientific attainments, the additions which he makes to this valuable work must be regarded with interest. But this interest is much increased by what appears in the prolegomena, viz. that this work comes out under the sanction of the present Professor of the Theory and Practice of Physic in the University of Pennsylvania; and that he adopts it as his text-book. Even more, we are informed that the editor has had access to some of Dr. Chapman's manuscript lectures, from which he has enriched his work, and from which, in two or three instances, he has given us extracts. The opportunity of learning the doctrines and precepts inculcated in the first medical school in our country, and, if the number of its pupils be a criterion, one of the first in the world, cannot be regarded with indifference.

But it must be known, that the doctrines referred to are not those which we have hitherto received from the Philadelphia school. Those were the doctrines of Rush; and, whether correct or not, we deem it certain, that his persuasive eloquence, and the force of his genius, caused them to be received with very little opposition during his life, by those who resorted to his lecture-room. Now that the voice of their author has ceased to resound on the ears of his pupils, these doctrines must rest on their own merits alone; and it remains to be seen which of them are placed on stable foundation, and which upon the personal influence of the celebrated Professor. Already it seems, that, under the sanction of the successor of Dr. Rush, there is a formal attempt to overthrow his most favourite principles, and almost to expose his system to derision.*

* We cannot believe that Dr. Rush would wantonly submit any thing from Dr. Cullen to derision; still less that Dr. Rush's successor would be guilty of such indecency to the illustrious dead. We are perfectly of opinion that nosology is liable, like all artificial arrangements of nature, and probably more than any other, to appear in a very disadvantageous attitude. Of the *unity* of diseases we shall say nothing till Dr. Rush's words and arguments are stated. We have already announced a very learned work on Nosology, which, if leisure permits, will appear in our next, and to which we promise the same candour as it is always our wish to show. Our opinion on that subject is very well known, but we trust it will not influence us. If it should, our readers will not question our good intentions, however they may doubt our impartiality.—*London Editor.*

"To

"To those, who, for the last fourteen years, have been conversant with the history of medicine in the United States, it is perfectly known, that a bold and persevering attempt was made, by the late Dr. Rush, to overthrow entirely Methodical Nosology, and erect, on its ruins, his favourite hypothesis of *the Unity of Disease.* Nor was he altogether unsuccessful in the pursuit of his enterprise. By a combination of popular and imposing qualities, superadded to an ascendancy derived from his station as a public teacher, he implanted in the minds of no inconsiderable portion of the physicians of America, a disbelief in the truth and value of classical medicine.

"To endeavour to counteract this evil, which, from the simplicity it appeared to have introduced into medical science, had become exceedingly seductive of indolent minds; to recall the prevalence of correct principles, touching the subdivisions and classification of disease; and, to restore to our profession the advantages of system, constitute the object of our Preliminary Discourse."—Preface to American edit. p. ix.

Accordingly, in the preliminary discourse, Dr. Caldwell points out the true basis of system and classification in science, viz. " affinity in some points, and dissimilarity in others;" and he shows the object and tendency of this classification. He illustrates its importance, and the benefits to be derived from it, by a reference to the various branches of natural history, and particularly to zoology. The only question is, whether diseases are susceptible of a similar arrangement. Dr. Rush thought that they were not, but Dr. Caldwell says that they are, since they have affinity in some points and dissimilarity in others; and subsequently he shows, that Rush himself had in effect a system of nosology. In his endeavours to establish the differences among diseases, Dr. Caldwell is led to consider the doctrine of unity of disease promulgated by Dr. Rush. On this doctrine were grounded the principal objections of this learned Professor to systematic nosology, and, if that can be proved to be untenable, the superstructure raised upon it must be deserted. The editor of this work contends, that the doctrine of the unity of disease implies *the unity of excitability* and *the unity of stimuli.* The editor himself seems to us not to distinguish sufficiently between sensibility and irritability, properties essentially different and perfectly distinct; but which, by Brown and his followers, are not distinguished at all. Without, however, availing himself of this distinction, the two properties being comprehended under the term excitability, Dr. Caldwell satisfactorily disproves the doctrines in question. In truth, we doubt exceedingly whether any physician ever lived, who, after one year's practice, verily believed that all stimuli produce the same effects, and that all parts of the body are affected in the same mode by the same stimuli.

In

In the conclusions at which Dr. Caldwell arrives, in regard to the doctrine of unity of disease, and in regard to the importance of a systematic arrangement of diseases, we fully accord with him; but we would not be understood as assenting to all the opinions expressed in his preliminary discourse. In the preference which he gives to Cullen's system of nosology over any other extant, he will, no doubt, be supported by the majbrity of voices; but, for ourselves, we cannot be so decided in preferring Cullen to Sauvages, not to bring into view the systems of any later writers. We shall fully agree, however, with Dr. Caldwell, in lamenting the imperfection of all the systems of nosology, and we think it would not be difficult to point out certain radical defects in them all. That they all have important defects, Dr. Caldwell is perfectly aware: in Cullen's system he states, that "its faults are numerous, and several of them conspicuous;" and he closes his discourse with "a brief consideration of some of the most important of them." On the criticisms which he makes, a few remarks must be offered on our parts.

First, he objects to Cullen's definition of fever, on account of its concluding clause, *sine morbo locali primario.** It was Dr. Cullen's object, by these words, to point out a distinction, long recognised by good observers, between the disease he was defining, called fever, or idiopathic fever, and those diseases included by Cullen in the same class under his order Phlegmasiæ. In the phlegmasiæ, when pure, there is first a local affection, viz. an inflammation, and then the system is affected by sympathy. The affection of the system in these cases has not one uniform character; sometimes it is marked by an affection of the sanguiferous system most especially, sometimes by an affection of the chylopoietic system, sometimes by an affection of the brain, and nervous system, &c. Similar varieties appear in the idiopathic fever (if the term may be employed), but without our being able to trace them to any peculiar causes. Now Dr. Caldwell contends, that, in the cases where we fail to discover any local disease, such a one must, nevertheless, exist; and he endeavours to prove it by showing, 1st, That it is possible for an adequate local cause to exist without exciting any sensation, and, therefore, if in an internal part, without being recognised. 2d. That "all febrile affections which we are capable of clearly tracing to their commencement, most certainly originate in a topical affection." 3d. That

* The definition is, Prægressis languore, lassitudine, et aliis debilitatis signis, pyrexia, sine morbo locali primario.

the the remote causes of fever cannot "gain access to the whole system at once; they must, therefore, attack locally, and afterwards extend their ravages on sympathetic principles."

It is probable our author would not believe that much was gained, should the two first arguments be admitted in all their force, unless the third was also admitted to be valid and to bear upon his point. It will suffice, then, to examine this third. Nor is it to be granted, at first, that all foreign causes, acting on the system, operate on parts and not on the whole. The parts primarily exposed to their action are the organs of sense, the skin, and the mucous membrane of the various passages having external outlets.* According to the doctrine in question, there must be some local affection on some of these parts in every case of fever, and the fever arises from sympathy of the whole system with this part.

We think that Dr. Caldwell will agree, that we have represented him fairly in the foregoing statement. But, if the argument be pursued, it seems to prove too much. For is any one disposed to deny that the affection of the system in pleurisy in peritonitis, &c. arises in these cases respectively from inflammation of the pleura, of the peritoneum, &c. But these are not the parts, on which the remote causes could have originally acted according to Dr. C.'s opinion; they are not the primary diseases; and, in accordance with the principles which he repeatedly advances, they must arise from sympathy, in consequence of disease in the skin, mucous membrane, &c. Turning, however, to those pages in the book before us, in which these opinions should have been expressed, we do not find them. The inference is, that the editor had not arrived at such conclusions.

The truth is, that the parts first acted upon, or first touched by external causes of disease, must be those before enumerated. But, causes applied to these parts produce disease in other and distant parts, without occasioning any evident disease in the parts first acted upon or touched; or if any thing, which can be called disease, be produced in these parts primarily, this disease is transient, and the continuance of the secondary affection is not dependent upon it. Thus, a temporary interruption of the cutaneous excretion may occasion an inflammation in the pleura. Four days afterwards, you may restore the excretion in any degree you please, the inflammation will continue in the pleura. So a temporary

* It is not necessary, for the purposes of the present discussion, to remark, that, in cases of solution of continuity, different surfaces are exposed to the action of external causes. Nor need we embarrass the question, by bringing into view the influence of the passions as causes of disease.

interruption interruption of the catamenia, or of the lochia, will occasion inflammation in the peritoneum, or in some other part; but the restoration of the uterine discharge will not remove the disease. The disease is produced *uno ictu;* and, being produced, it goes through its stages without reference to its cause. At least, after the disease is once fairly established, this is the case.

Now we conceive, that on the same principles may be explained the occurrence of what has been called idiopathic fever; and, that when it is said, that this occurs *without a primary local disease*, it is not meant to deny, that some unusual impression has been made on some point in the body. Such an unusual impression may, philosophically considered, constitute a disease; but nothing is considered a disease in the common view of the subject, of which we have not some palpable evidence. In this case, it is meant to deny, that there exists any local disease, on which the affection of the system depends for its maintenance or continuance. It is meant to deny, that the affection of the system stands on the same ground, as that which follows common inflammation, and which may be removed by removing the inflammation. It is true, that idiopathic fever is sometimes removed at an early period, by applications made to the stomach, and sometimes by applications made to the skin. But this does not prove, that the disease depends, in these cases, on the local affections of the stomach or skin.

Believing as we do, that idiopathic fever, (febris of Cullen,) is an affection of the whole system *sui generis*, altogether distinct in its nature from the sympathetic affections produced by inflammation, we could not pass unnoticed the attempt from so respectable a quarter, to confound these different affections. But, to expose the whole ground of argument on these topics would lead us too far.

There is another subject, which is taken up in the preliminary discourse, and to which the editor frequently refers in his notes, in which we do not perfectly accord with him. This is the subject of contagion. In condemning the loose and indistinct views, which have too often been entertained by others, he, perhaps, goes to the opposite extreme, and limits too much the evidence to be admitted in proof of contagion. The small-pox may be communicated through the atmosphere by effluvia, and, likewise, by bringing the fluid or dried virus, produced on one subject, into contact with the naked fibre of another. In examining this virus, it is not found to have any sensible or chemical properties, by which it can be distinguished from matter produced in the human subject under some other circumstances. The property of reproducing, in a fresh subject, the same disease by which it has has been formed in another, is learnt only by experience. There are several other circumstances learned by observation and experience, in regard to this disease. 1. When a person has once undergone the disease, he is incapable of undergoing it again. There are, however, some rare exceptions to this; and certain local effects may be repeatedly produced by the virus on the same subject. 2. This disease may be produced by an exceedingly small quantity of the virus, as perfectly as by a large quantity. 3. This disease may be made to occur at all seasons and in all climates. Under this head, however, it is to be remembered, that, in places where it always exists, it is in certain years and seasons vastly more prevalent, than in other years and seasons. Alas, it is very different in its degree of severity at different periods. 4. It occurs at a certain fixed period, that is, about fourteen days after exposure, when produced by effluvia. After inoculation, it also affects the system at a certain period; but this is shorter than when it is communicated by effluvia. Neither of these periods is of precisely the same length in all cases, varying from two to four days, without any obvious cause. In consequence of the intervention of other diseases, they may be protracted much longer. 5. We do not know the origin of this disease; but we have reason to believe, that it has not, for several ages, been produced in any other way than by contagion.

We know, then, that small-pox is contagious; and we know, that the laws, just enumerated, exist in respect to this disease. But we do not know, that these laws are necessarily connected with its power of re-producing itself by contagion. We are not, therefore, to deny the property of contagion to any other disease, because the same laws are not found to exist in respect to such disease. If it was certain, that in regard to the mode of communication, or in regard to all or any of the laws enumerated, all other contagious diseases must resemble small-pox, the difficulty of deciding whether a disease is contagious would be very much diminished.

It certainly is possible, that a disease originating on this day, for the first time, may be contagious. It certainly is possible, that diseases frequently originating *de novo*, and in various places, may be contagious. It certainly is possible, that a disease may be contagious only among persons, who have been predisposed to it by some atmospheric influence. It is true, that a good deal of circumstantial evidence is necessary to satisfy us of the contagious character of diseases of the descriptions now given. But the possibility of their having such a character cannot be denied. On the other hand, in proportion as a disease is subject to the laws enumerated as applying to small-pox, we are more ready to admit evidence in favour of its being contagious.

What, then, shall be admitted as conclusive evidence of the contagious power of a disease? We know not that one kind of evidence alone is to be admitted. Inoculation makes the evidence demonstrative; but we must not refuse to be satisfied by any evidence short of this. If an unusual disease should prevail among us, and it should be made clear, that the first person affected with the same disease had recently arrived from some other district, in which that disease had been prevalent before his departure, there would arise a strong suspicion, that this disease was contagious. Should it appear only among those, who had been, in some way, exposed to the sick, this suspicion would become more strong. Should now a considerable portion of the healthy inhabitants be removed to a neighbouring place, and should all intercourse between them and the other inhabitants be prevented, and should those who had thus removed escape from the disease, while it continued to prevail among the others, the evidence would be nearly irresistible. It is true, that even here it would be possible for us to be misled. The evidence would not be so strong as that arising from inoculation. Yet it would be such as rightfully to influence our conduct. We should in such a case take the same precautions as if we were perfectly sure of the contagious power of the disease.*

To this subject, the evidence of contagion, there are repeated references in the notes to the work before us, as well

---

* *Note of the English Editor.*—There appears to us some falacy, if not danger, in the positions contained in the above paragraph, because the converse of the proposition, as well as the proposition itself, is liable to objection. 1st, Should the disease appear only among those who have had intercourse with diseased subjects, it will be necessary to inquire, whether they have not had intercourse with those who arrived from the place in which the disease originated; and, if so, the apparel may convey the morbific effluvia; and, if none in this second district were infected, but those who had approached the strangers, then we should not call the disease contagious, but by some other description, as long as we consider the small-pox contagious. It has been urged, that there may be various *kinds* and degrees of contagion. As to the first, we have only to answer, that two *kinds* of any thing should either have two names, or at least some term to distinguish them. As to the degrees, we well know that they vary in the small-pox at different times. If, therefore, the mode of communicating a disease is similar in other respects, the degree is of less importance, because the mode of prevention must be similar.

as in the preliminary discourse, and the editor seems to limit much more than we have done the evidence which is to be deemed satisfactory.

At page 141, in the same volume, the subject of quarantine is adverted to, and the wish is expressed, that the matter may be more fully inquired into, and the necessity of the practice considered impartially. It is certain, that the quarantine laws are the source of great inconvenience. If they are useless, the burden should be removed. It is in this country that the subject should be investigated; for, at present, we not only suffer at home by this practice, but we give occasion, by this very circumstance, to many foreign powers to subject our trade to great embarrassment abroad, in cases where it certainly is not necessary.

At page 215, vol. i. we learn that Dr. Caldwell adopts the opinion originally proposed by Dr. Lubbock and Mr. Allen; or rather, if we remember right, by some Italian physician first of all.—This is, that the impetus of the blood through the vessels of an inflamed part is diminished, not increased, and that the vessels are debilitated. It does not seem evident to us, that the impetus is necessarily either increased or diminished during inflammation. The enlargement of the vessels is not to be explained mechanically, but by the power of elongation possessed by the fibres of their muscular coats, which has been taught by Hunter and by Barthez. But that it is essential for vessels to be debilitated, in order to the performance of the new and extraordinary functions of inflammation—this is hard to believe. In regard to Dr. Wilson's experiments, we are satisfied that they are not worthy of any regard on this point.

While considering the process of suppuration, the editor claims for one of our countrymen, an honour, which has, we believe, been commonly thought to lie between the celebrated De Haen and John Hunter. We have never seen the dissertation by Dr. Morgan; but the British physicians will ask, whether the doctrine it asserts had not been already promulgated by Hunter in 1765.*

---

* We are glad to see this answer from another quarter. Our known partiality might have been suspected. We have often heard with regret, from the disciples of one of the London schools, that a similar doctrine has been promulgated there. Whoever compares either De Haen or Morgan with Hunter, will require no other arguments in favour of the latter.—Add to this, Mr. Hunter gave public lectures in the year 1755.—*English Editor.*

